# Assessment of the Inter-Frequency Amplitude Ratio (1000/500 Hz) in cVEMP and oVEMP for the Diagnosis of Ménière’s Disease

**DOI:** 10.3390/audiolres14060093

**Published:** 2024-12-20

**Authors:** Sacha Drabkin, Antonino Maniaci, Mario Lentini, Giannicola Iannella, Sophie Tainmont, Christophe Lelubre, Quentin Mat

**Affiliations:** 1 Department of Otorhinolaryngology, Centre Hospitalier Universitaire de Charleroi, Chaussée de Bruxelles 140, 6042 Charleroi, Belgium; 2 Otology Study Group, Young Otolaryngologists-International Federation of Otorhinolaryngological Societies, 13005 Paris, France; 3 Department of Medicine and Surgery, University of Enna Kore, 94100 Enna, Italy; 4 Department of Surgery, Asp 7 Ragusa, Modica Hospital, 97100 Ragusa, Italy; 5 Department of ‘Organi di Senso’, University “Sapienza”, 00185 Rome, Italy; 6 Department of Internal Medicine, Centre Hospitalier Universitaire de Charleroi, Charleroi, Chaussée de Bruxelles 140, 6042 Charleroi, Belgium; 7 Faculty of Medicine and Pharmacy, University of Mons (UMons), 7000 Mons, Belgium

**Keywords:** Ménière’s disease, c-VEMP, o-VEMP, interfrequency amplitude ratio, IFAR

## Abstract

**Objectives**: to retrospectively evaluate the clinical relevance of the 1000/500 Hz inter-frequency amplitude ratio (IFAR) in cervical and ocular vestibular evoked myogenic potentials (cVEMPs and oVEMPs) in patients with unilateral definite Ménière’s disease (MD) to identify the pathological ear. **Method**: cVEMPs and oVEMPs results obtained at 500 Hz and 1000 Hz were retrospectively analyzed in 28 patients with unilateral definite MD. 1000/500 Hz IFAR were calculated and compared for each ear. Spearman correlation tests between patient age and 1000/500 Hz IFAR were also performed. **Results**: No significant difference was observed between the 1000/500 Hz IFAR calculated in both pathological and healthy ears when the cVEMPs were performed (*p* = 0.74; Wilcoxon signed-rank test). 1000/500 Hz IFAR results obtained in healthy and pathological ears were also not different for oVEMPs (*p* = 0.73; Wilcoxon signed-rank test). Analysis of modified 1000/500 Hz IFARs for healthy and pathological ears showed no difference in both cVEMPs and oVEMPs (*p* = 0.44; *p* = 0.95, respectively; Wilcoxon signed-rank test). There was a significant positive correlation between IFARs, modified IFARs, and patient age for cVEMPs (*p* = 0.017; *p* = 0.012, respectively, Spearman’s correlation test). A significant positive correlation was also found between modified IFARs and the subject age in oVEMPs (*p* = 0.019, Spearman’s correlation test). **Conclusions**: We did not observe any significant increase of 1000/500 Hz IFARs and 1000/500 Hz modified IFARs in ears affected by definite MD compared to healthy ears. Moreover, our research suggests that the age of the participants may influence IFAR results, which may lead to misdiagnosis in the elderly. It is, therefore, essential to conduct further prospective studies in larger cohorts, stratifying results by participant age, to better understand the role of 1000/500 Hz IFAR values in the diagnosis of MD.

## 1. Introduction

Definite Ménière’s disease (MD) is a vestibular disorder that significantly impairs quality of life [1]. It is characterized by episodic vertigo associated with low to medium sensorineural hearing loss, and fluctuating aural symptoms (hearing, tinnitus, and/or fullness) in the affected ear. Episodes of vertigo can last from 20 min to 12 h. Diagnosis is therefore based on clinical and audiometric criteria published by the Bárány Society [2].

Endolymphatic hydrops (EH) is associated with this disease and can now be seen 4 h after a single intravenous dose of contrast agent with a 3 Tesla MRI of the inner ear [3]. However, EH cannot be seen in all cases of MD on MRI and, conversely, it has also been reported in other pathologies such as vestibular schwannomas, recurrent peripheral vestibulopathy (RPV), perilymphatic fistulas and inner ear malformations [3,4,5,6,7]. Therefore, identifying a specific and sensitive paraclinical vestibular test for MD is currently a major challenge, especially in the absence of auditory symptoms.

Vestibular evoked myogenic potentials (VEMPs) are short-latency electromyographic responses triggered by intense acoustic, vibratory, or galvanic stimuli [8,9,10]. The two main clinical subtypes are cervical VEMP (c-VEMP) and ocular VEMP (o-VEMP) [8,9,10].

C-VEMPs are recorded on the sternocleidomastoid muscle (SCM) [8,9]. They are generated by the activation of the saccule, course along the inferior vestibular nerve, and induce an inhibitory reflex in the ipsilateral SCM [9,10,11]. They are used to test the integrity of the ipsilateral vestibulocollic pathway [12]. O-VEMPs are generated after activation of utricular receptors and course along the superior vestibular nerve to the contralateral inferior oblique muscle, thus activating it. They are used to assess the contralateral vestibulo-ocular pathway [9,10,13].

Typically, VEMPs are recorded using a 500 Hz tone burst stimulus, as this tends to elicit the best responses and a high response rate in young adults. However, higher amplitudes and response rates have been found at 1000 Hz in the case of MD [9,14].

A recent development has been the introduction by some authors of the concept of inter-frequency amplitude ratio (IFAR), defined as the ratio of the peak-to-peak amplitude recorded at 1000 Hz to the peak-to-peak amplitude recorded at 500 Hz in the same ear when c-VEMP and o-VEMP are performed. This may help identify patients with definite MD [15].

The main objective of this study was to retrospectively evaluate the clinical relevance of 1000/500 Hz IFAR in vestibular evoked myogenic potentials (c-VEMP and o-VEMP) to identify the pathological ear in patients with unilateral definite MD.

## 2. Materials and Methods

Patients:

Our retrospective study was performed at the University Hospital of Charleroi in Belgium. A total of 28 patients with unilateral definite MD (12 men, 16 women, median age = 66.5 years, Q1–Q3= 49–71 years, extremes = 29–87 years), selected based on the Bárány Society criteria, were included. All patients had a complete vestibular assessment during the diagnostic work-up, including bithermal caloric testing, video head impulse test (vHIT), c-VEMPs and o-VEMPs. Pure-tone liminar audiometry was also performed with a TDH 39 headphone and B71 bone vibrator in a sound-treated audiometric test booth. Pure tone averages (PTA) were then calculated for MD-affected ears and healthy ears. PTA was calculated from the average of the air-conducted hearing thresholds obtained from 500, 1000, 2000, and 4000 Hz during pure tone audiometry.

Patients with bilateral MD or any other condition that could alter air-conducted VEMP results were excluded. Therefore, patients with the following conditions were excluded from the study:

- Outer and/or middle ear malformations that could affect sound transmission to the ipsilateral or contralateral inner ear (e.g., tympanic membrane perforation, otosclerosis, etc.).
- Malformation of the ipsilateral or contralateral inner ear
- Third window syndrome (e.g., superior semicircular canal dehiscence syndrome and large vestibular aqueduct)
- Afferent neural pathway disorder (e.g., vestibular schwannoma, history of vestibular neuritis, multiple sclerosis, stroke, etc.).
- Cervical problems (cervical spinal cord compression, herniated disk, history of cervical arthrodesis, or other cervical surgery, etc.).
- Oculomotor anomalies.
- Neuromuscular disorders.
- Patients for whom no VEMP testing had been performed at the time of assessment were also excluded.

Therefore, each of the patients included benefitted from a brain MRI and a cone beam CT-scan of the temporal bone. These also allowed for the exclusion of certain pathologies that could mimic a unilateral MD (inner ear malformation, endolymphatic sac tumor and vestibular schwannoma).

VEMP recording:

To record VEMPs, participants were seated inside a soundproof, faradized booth. Both ether and Nuprep gel were utilized to clean the skin before applying the electrodes. Insert earphones were inserted in each ear (monaural testing) and the system was calibrated in accordance with ISO 389-6.

The Eclipse EP15 module was used to record vestibular evoked myogenic potential after delivering sound stimuli.

For c-VEMPs, the active electrode was placed on the middle third of the SCM, ipsilaterally to the sound stimulation. The reference electrode was placed on the manubrium and the ground electrode was placed in the center of the forehead. Participants were asked to turn their head so that it was contralateral to the sound stimulation throughout the recording. The contraction level for the SCM was monitored before sound stimulation using the surface electrodes used to record the c-VEMP responses. The accepted pre-stimulation contraction level ranged from 50 microV to 150 microV. Electromyographic activity was displayed on a screen to monitor muscle contraction in the patient’s ipsilateral SCM and adjust contraction if necessary.

To record o-VEMPs, a “belly-tendon” electrode montage was used: the active electrode was positioned on the lower eyelid, contralateral to the stimulated side, slightly offset outward from the center of the eye. The reference electrode was placed on the medial canthus contralateral to stimulation, and the ground electrode was placed on the forehead.

Each ear was tested by tone bursts at 500 Hz and 1000 Hz at an intensity level of 95 dB nHL for c-VEMPs and 100 dB nHL for o-VEMPs. Each stimulus lasted 6 ms (rise, fall, and plateau times of 2-2-2 ms) and was repeated 200 times for one acquisition for c-VEMP and 100 times for o-VEMP. The repetition rate was 5.1/s with a rarefaction polarity and a band-pass filter of 10 to 750 Hz for c-VEMP, and of 1 to 1000 Hz for o-VEMP. Electrical activity was recorded from 20 ms before to 80 ms after stimulus onset.

The following variables of interest were recorded: peak-to-peak N1-P1 amplitude for o-VEMP, corrected P1-N1 amplitude for c-VEMP, and P1, and N1 latencies for c-VEMP and o-VEMP. A response was considered valid only if a biphasic wave was identified at the expected latencies for a tone burst, with an amplitude greater than the noise recorded prior to stimulation. If no amplitude was recorded, it was considered absent. Each recording was repeated twice, and the two measured values were averaged for analysis.

Statistical analysis:

The Shapiro–Wilk test was used to test for normality distribution. Therefore, comparisons of pure tone averages obtained in pathological ears and healthy ears were performed with a paired samples *t*-test. The following analyses were performed using nonparametric tests. The Wilcoxon signed-rank test was used to compare the continuous variables between healthy and pathological ears. Correlation analyses were performed using Spearman’s correlation test. A *p*-value of less than 0.05 was considered statistically significant. Statistical analyses were performed using JASP Team (2024) software (JASP (Version 0.18.3)).

The ethics committee of Charleroi has accepted and approved the study protocol (ethical approval code: B3252023000066).

## 3. Results

### 3.1. Descriptive Statistics

The distribution of the variables of interest of c-VEMPs and o-VEMPs is shown in Table 1.

### 3.2. Inferential Statistics

#### 3.2.1. Comparison of PTA Obtained in Ears with MD and Healthy Ears

**Figure 1:**
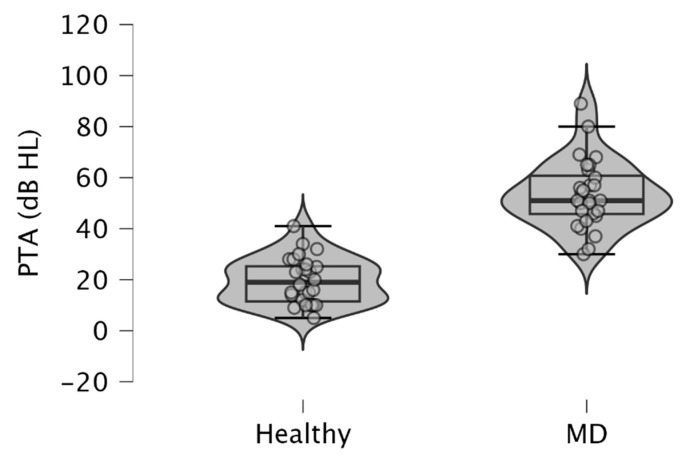
Violin plots and box plots showing pure tone averages (PTA) obtained in ears with MD and healthy ears. The pure tone average was calculated from the average of the air-conducted hearing thresholds obtained from 500, 1000, 2000, and 4000 Hz during pure tone audiometry. Healthy, healthy ears; MD, affected ears with Ménière’s disease.

Since MD is characterized by low to medium sensorineural hearing loss, we observed higher PTA in MD-affected ears (mean ± SD = 53.54 ± 13.38 dB HL), than in healthy ears (mean ± SD = 19.61 ± 8.95 dB HL) (*p* < 10^−3^; paired samples *t*-test) (Figure 1). These results were obtained from pure tone audiometry performed on the same day that c-VEMPs and o-VEMPs were realized on a patient.

#### 3.2.2. 1000/500 Hz IFAR and c-VEMPs

**Figure 2:**
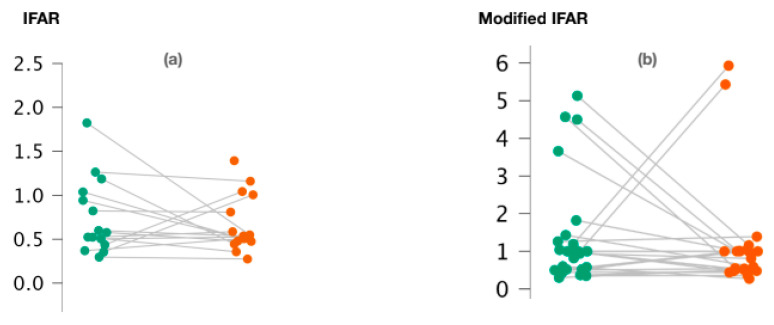
Variation of 1000/500 Hz IFAR values (**a**) and 1000/500 Hz modified IFAR values (**b**) according to the ear studied in c-VEMPs.

We observed no significant difference between 1000/500 Hz IFARs calculated for healthy and pathological ears when the cVEMPs were performed (*p* = 0.74; Wilcoxon signed-rank test) (Figure 2a). We also calculated “modified” 1000/500 Hz IFARs by replacing zero amplitude values with a 1. Similarly, with regard to c-VEMPs, no significant difference was observed between the two groups (*p* = 0.44; Wilcoxon signed-rand test) (Figure 2b).

Figure 2a,b represents the scatter plots of the 1000/500 Hz IFAR and modified IFAR values calculated for healthy (green) and affected (red) ears, respectively. The intraindividual values are connected together by a line. C-VEMP, cervical vestibular evoked myogenic potential; Hz, Hertz; IFAR, inter-frequency amplitude ratio.

#### 3.2.3. 1000/500 Hz IFAR and o-VEMPs

**Figure 3:**
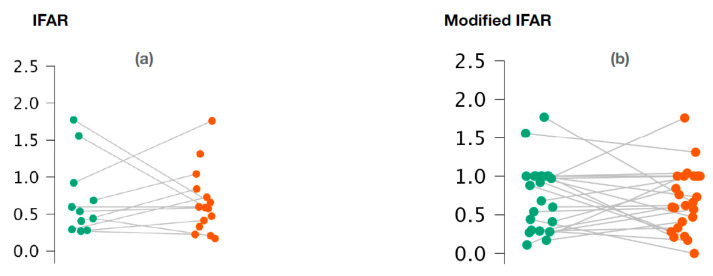
Variation of 1000/500 Hz IFAR values (**a**) and 1000/500 Hz modified IFAR values (**b**) according to the ear studied in o-VEMPs.

With regard to o-VEMPs, no statistically significant difference was observed between the affected ears and the healthy ears for 1000/500 Hz IFARs (*p* = 0.73; Wilcoxon signed-rank test) (Figure 3a). We also did not observe any difference with the 1000/500 Hz modified IFARs (*p* = 0.95; Wilcoxon signed rank) (Figure 3b).

Figure 3a,b represents the scatter plots of the 1000/500 Hz IFAR and modified IFAR values calculated for healthy (green) and affected (red) ears, respectively. The intraindividual values are connected together by a line. O-VEMP, ocular vestibular evoked myogenic potential; Hz, Hertz; IFAR, inter-frequency amplitude ratio.

#### 3.2.4. 1000/500 Hz IFAR and Age

We performed Spearman’s correlation test to assess the relationship between age and 1000/500 Hz IFARs. We obtained a positive and statistically significant correlation between IFARs and participants’ age (*p* = 0.017, r = 0.425, Spearman’s correlation test) when the c-VEMP test was performed. For modified IFARs, a positive correlation with participant age was also observed (*p* = 0.012, r = 0.352, Spearman’s correlation test).

**Figure 4:**
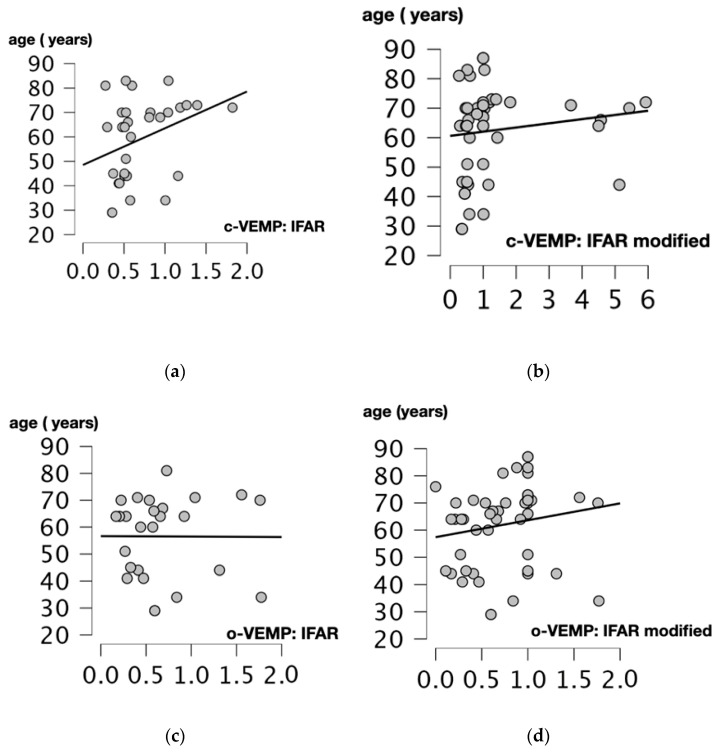
Scatter plots of Spearman correlation coefficient between the age (years) and 1000/500 Hz IFAR for c and o-VEMPs. Correlations between: (**a**) Age and 1000/500 Hz IFARs obtained when the cVEMPs were performed (*p* = 0.017, r = 0.425). (**b**) Age and 1000/500 Hz modified IFAR obtained when the c-VEMPs were performed (*p* = 0.012, r = 0.352). (**c**) Age and IFAR 1000/500 Hz obtained when the o-VEMPs were performed (*p* = 0.756, r = 0.06). (**d**) Age and modified IFAR 1000/500 Hz obtained when o-VEMPs were performed (*p* = 0.019, r = 0.332). C-VEMP, cervical vestibular evoked myogenic potential; o-VEMP, ocular vestibular evoked myogenic potential; Hz, Hertz; IFAR, inter-frequency amplitude ratio.

For the o-VEMP test, there was no significant correlation between 1000/500 Hz IFARs and subject age (*p* = 0.756, r = 0.06, Spearman’s correlation test). The only statistically significant positive correlation was between modified IFARs and patient age (*p* = 0.019, r = 0.332, Spearman’s correlation test) (Figure 4).

## 4. Discussion

MD is diagnosed based on clinical and audiometric criteria [2]. In the absence of auditory symptoms, the diagnosis can be challenging. To date, there is no paraclinical vestibular test that can accurately identify this condition. Recently, Kim Lee et al. proposed a 1000/500 Hz IFAR-based analysis of VEMP to diagnose MD [15]. Indeed, while young and middle-aged healthy subjects usually respond better to VEMP testing at 500 Hz, several studies have shown that patients with MD have higher VEMP amplitudes and response rates at higher stimulation frequencies [14,15]. Rauch et al. (2004) suggested that pathological inner ears responsible for MD have a maximum amplitude when VEMPs were performed with an air conducted stimulus at 1000 Hz [16,17]. Although the exact reason for this change in frequency remains unclear, some authors attributed it to the increased pressure in the endolymphatic space of the saccule and utricle (endolymphatic hydrops), which can affect the stiffness of the system and the resonance frequency of the otolith organs [18].

In our study, we found no statistically significant increase in IFARs in the pathological ears compared to the healthy ears when c-VEMP and o-VEMP were performed. Nor did we observe any tendency toward an increase in IFARs in pathological ears. Indeed, the calculated IFARs sometimes increased, decreased, or remained unchanged compared to the healthy ears.

The literature on IFAR calculation in MD is scant. Moreover, different approaches to IFAR calculation have been reported, and the method used in different studies is not always clearly explained. For example, Maxwell et al. replaced null amplitude values by artificially modifying them with a 1 to obtain a larger number of calculable IFARs (=modified IFARs) [19]. In practice we did not always obtain responses at 500 Hz and 1000 Hz in the same ear (either because the otolith organs had been damaged by the disease or because of the natural aging of the organs). As a result, we were only able to calculate IFARs in 40-60% of the c-VEMP and o-VEMP cases (see Table 1). The response percentage of the VEMP and IFARs reported in the literature varies from 10 to 80% in the affected ears of patients with MD [17,20,21,22]. Therefore, our aim was to determine whether the transformation of IFARs into modified IFARs would lead to an increase in modified IFARs in pathological ears compared to healthy ears. However, we did not observe this effect for either c-VEMP or o-VEMP tests. Other techniques for modifying IFARs have been proposed. These, however, are even further removed from the rationale that endolymphatic hydrops produces better responses at 1000 Hz than at 500 Hz [23]. Moreover, we did not observe significantly higher amplitudes at 1000 Hz than at 500 Hz in either healthy or pathological ears when c-VEMP and o-VEMP tests were performed (Table 1). It is also worth noting that most studies evaluating 1000/500 Hz IFARs when c-VEMPs were performed analyzed raw rather than corrected amplitude values [19,21,23]. Raw amplitude values have been shown to increase with SCM contraction, potentially altering amplitude values at 500 Hz and 1000 Hz independently of endolymphatic hydrops. Therefore, it is necessary to normalize c-VEMP amplitudes according to the pre-stimulus SCM contraction and to analyze only the corrected amplitudes [9,24].

Recently, some authors showed that increasing age in healthy subjects was associated with increased 1000/500 Hz IFARs for VEMPs and better response rates at 1000 Hz rather than 500 Hz. This increase in the resonance frequency of otolith organs in the elderly for 1000 Hz can be explained by a reduction in the otolith membrane mass due to loss of otoconia as a result of progressive degeneration [25,26,27]. In contrast to other studies that have examined the utility of 1000/500 Hz IFARs in the detection of MD, our study population is relatively old (66 years, Q1 = 49 years, Q3 = 71 years, range: 29 and 87 years). It is possible that the advanced age of the participants alone generates an increase in 1000/500 Hz IFAR values bilaterally, and that this increase precludes the identification of any differences that may have been generated by endolymphatic hydrops [16,21,23,28]. We conducted correlation tests between the age of the participants and the results of the 1000/500 Hz IFAR values and the modified 1000/500 Hz IFAR values. We observed a significant positive correlation between the age of the participant and the 1000/500 Hz IFAR values and between the age of the participants and 1000/500 Hz modified IFAR values for cVEMP. However, with regard to oVEMP, we observed only a significant correlation with 1000/500 Hz modified IFARs values. The lack of correlation between patient age and 1000/500 Hz IFAR values for oVEMP may be explained by the low number of 1000/500 Hz IFAR values obtained. Consequently, the detection of MD in an elderly population by means of 1000/500 Hz IFAR values or modified 1000/500 Hz IFAR values seems to be an insufficiently sensitive test since it already depends on the age of the participants [25,26,27,28]. These observations are consistent with those of Singh et al. (2023) and Jha et al. (2024) who evaluated the utility of 1000/500 Hz IFAR values in the diagnosis of MD in comparison with a control group of similar average age [25,27]. They reported lower sensitivity and specificity than studies comparing 1000/500 Hz IFARs with healthy controls who were younger than the affected population, which may be a source of bias. Different cut-off values, ranging from 0.7 to 1.2, have been proposed for the identification of MD [29]. We found several modified 1000/500 Hz IFAR values higher than 0.7 in our population in both healthy and pathological ears, highlighting a possible age effect on our results. However, it cannot be ruled out that the analysis of the 1000/500 Hz IFAR values and modified 1000/500 Hz IFAR values may be of interest for the detection of definite MD occurring in a young population.

Finally, our study has some limitations that should be highlighted. First, the small number of patients included in this study could prevent us from obtaining significance in statistical tests. However, the analysis of scatter plots of the 1000/500 Hz IFAR and 1000/500 Hz modified IFAR values in cVEMPs and oVEMPs does not seem to show any trend towards higher values in the affected ears. In addition, the retrospective nature of this study could limit our results. Indeed, the duration of MD symptoms was not always reported when analyzing patient records and the duration of MD could influence the results of cVEMPs and oVEMPs [20,30]. However, the main interest of calculating 1000/500 Hz IFAR should be to obtain an early diagnosis of the disease and especially when there are no auditory symptoms yet. Therefore, further prospective analyses of larger cohorts of patients with unilateral MD, with stratification of results according to the age of the participants, are needed to confirm these results.

## 5. Conclusions

We did not observe any significant increase of 1000/500 Hz IFARs and 1000/500 Hz modified IFARs in ears affected by definite MD compared to healthy ears. Moreover, our research suggests that the age of the participants may influence IFAR results, which may lead to misdiagnosis in the elderly. It is therefore essential to conduct further prospective studies in larger cohorts, stratifying results by participant age, to better understand the role of 1000/500 Hz IFAR values in the diagnosis of MD.

## Figures and Tables

**Table 1:** Descriptive analysis of amplitudes, latencies and 1000/500 Hz IFARs obtained in both pathological and healthy ears when the c-VEMP and o-VEMP were performed at 500 Hz and 1000 Hz.

	Affected Ears		Healthy Ears	
	cVEMP	oVEMP	cVEMP	oVEMP
**500 Hz**				
Response percentage	75% (21/28)	60.7% (17/28)	71% (20/28)	64.2% (18/28)
Median amplitude (Q1–Q3)	0.28 (0.22–0.7)	3.47 µV (1.14–5.33) µV	0.48 (0.27–0.92)	3.49 µV (1.96–8.53) µV
Median P1 latency (ms) (Q1–Q3)	14 (13–16)	15 (14–17)	13.7 (13–14.2)	15.12 (14.4–16)
Median N1 latency (ms) (Q1–Q3)	22.1 (21–24.3)	10.3 (9.6–11.1)	22 (21.5–23.4)	10.1 (9.7–10.6)
**1000 Hz**				
Response percentage	53.5% (15/28)	39.2% (11/28)	57.1% (16/28)	53.5% (15/28)
Median amplitude (Q1–Q3)	0.29 (0.24–0.40)	1.77 µV (0.92–4.69) µV	0.36 (0.25–0.51)	2.4 µV (1.79–3.30) µV
Median P1 latency (ms) (Q1–Q3)	11.8 (11–14.4)	15.3 (13.33–16.2)	12.1 (11.3–12.6)	15 (13–16.5)
Median N1 latency (ms) (Q1–Q3)	20.6 (18.7–22.6)	10.1 (9.5–10.2)	21.2 (20–22.2)	9.5 (9.1–11.3)
**1000/500 Hz IFAR**				
Response percentage	53.7% (15/28)	39.2% (11/28)	57.1% (16/28)	53.5% (15/28)
Median 1000/500 Hz IFARs (Q1–Q3)	0.57 (0.47–0.99)	0.53 (0.35–0.8)	0.53 (0.48–0.86)	0.58 (0.37–0.78)

cVEMP, cervical vestibular evoked myogenic potential; oVEMP, ocular vestibular evoked myogenic potential; Hz, Hertz; ms, millisecond; μV, microvolt; Q, quartile; IFAR, inter-frequency amplitude ratio.

## Data Availability

The data presented in this study are available on request from the corresponding author due to privacy considerations.

## References

[1] Molnár A., Maihoub S., Mavrogeni P., Tamás L., Szirmai Á. (2022). Depression scores and quality of life of vertiginous patients, suffering from different vestibular disorders. Eur. Arch. Otorhinolaryngol..

[2] López-Escámez J.A., Carey J. (2015). Diagnostic criteria for Menière’s disease. J. Vestib. Res..

[3] Attyé A., Eliezer M., Boudiaf N., Tropres I., Chechin D., Schmerber S., Dumas G., Krainik A. (2017). MRI of endolymphatic hydrops in patients with Meniere’s disease: A case-controlled study with a simplified classification based on saccular morphology. Eur. Radiol..

[4] Attyé A., Dumas G., Troprès I., Roustit M., Karkas A., Banciu E., Pietras J., Lamalle L., Schmerber S., Krainik A. (2015). Recurrent peripheral vestibulopathy: Is MRI useful for the diagnosis of endolymphatic hydrops in clinical practice?. Eur. Radiol..

[5] Attyé A., Eliezer M., Galloux A., Pietras J., Tropres I., Schmerber S., Dumas G., Krainik A. (2017). Endolymphatic hydrops imaging: Differential diagnosis in patients with Meniere disease symptoms. Diagn. Interv. Imaging.

[6] Naganawa S., Kawai H., Sone M., Nakashima T., Ikeda M. (2011). Endolympathic hydrops in patients with vestibular schwannoma: Visualization by non-contrast-enhanced 3D FLAIR. Neuroradiology.

[7] Karch-Georges A., Veillon F., Vuong H., Rohmer D., Karol A., Charpiot A., Meyer N., Venkatasamy A. (2019). MRI of endolymphatic hydrops in patients with vestibular schwannomas: A case-controlled study using non-enhanced T2-weighted images at 3 Teslas. Eur. Arch. Otorhinolaryngol..

[8] Colebatch J.G., Halmagyi G.M.N. (1994). Myogenic potentials generated by a click-evoked vestibulocollic reflex. J. Neurol. Neurosurg. Psychiatry.

[9] Rosengren S.M., Colebatch J.G., Young A.S., Govender S., Welgampola M.S. (2019). Vestibular evoked myogenic potentials in practice: Methods, pitfalls and clinical applications. Clin. Neurophysiol. Pract..

[10] Hegemann S.C.A., Bery A.K., Kheradmand A. (2024). Focused Update on Clinical Testing of Otolith Organs. Audiol. Res..

[11] Murofushi T., Curthoys I.S. (1997). Physiological and anatomical study of click-sensitive primary vestibular afferents in the guinea pig. Acta Otolaryngol..

[12] Curthoys I.S., Vulovic V., Burgess A.M., Sokolic L., Goonetilleke S.C. (2016). The response of guinea pig primary utricular and saccular irregular neurons to bone-conducted vibration (BCV) and air-conducted sound (ACS). Hear. Res..

[13] Govender S., Dennis D.L. (2015). Vestibular evoked myogenic potentials (VEMPs) evoked by air- and bone-conducted stimuli in vestibular neuritis. Clin. Neurophysiol..

[14] Rauch S.D., Zhou G., Kujawa S.G., Guinan J.J., Herrmann B.S. (2004). Vestibular evoked myogenic potentials show altered tuning in patients with Meniere’s disease. Otol. Neurotol..

[15] Kim-Lee Y., Ahn J.H., Kim Y.K., Yoon T.H. (2009). Tone burst vestibular evoked myogenic potentials: Diagnostic criteria in patients with Meniere’s disease. Acta Otolaryngol..

[16] Singh N.K., Barman A. (2016). Frequency–amplitude ratio of ocular vestibular-evoked myogenic potentials for detecting Meniere’s disease: A preliminary investigation. Ear Hear..

[17] Taylor R.L., Zagami A.S., Gibson W.P., Black D.A., Watson S.R., Halmagyi M.G., Welgampola M.S. (2012). Vestibular evoked myogenic potentials to sound and vibration: Characteristics in vestibular migraine that enable separation from Meniere’s disease. Cephalalgia.

[18] Morita N., Kariya S., Deroee A.F., Cureoglu S., Nomiya S., Nomiya R., Harada T., Paparella M.M. (2009). Membranous labyrinth volumes in normal ears and Meniere’s disease: A three-dimensional reconstruction study. Laryngoscope.

[19] Maxwell R., Jerin C., Gurkov R. (2017). Utilisation of multi-frequency VEMPs improves diagnostic accuracy for Meniere’s disease. Eur. Arch. Otorhinolaryngol..

[20] Taylor R.L., Wijewardene A.A., Gibson W.P., Black D.A., Halmagyi G.M., Welgampola M.S. (2011). The vestibular evoked-potential profile of Ménière’s disease. Clin. Neurophysiol..

[21] Sandhu J.S., Low R., Rea P.A., Saunders N.C. (2012). Altered frequency dynamics of cervical and ocular vestibular evoked myogenic potentials in patients with Ménière’s disease. Otol. Neurotol..

[22] Winters S.M., Berg I.T., Grolman W., Klis S.F. (2012). Ocular vestibular evoked myogenic potentials: Frequency tuning to air-conducted acoustic stimuli in healthy subjects and Ménière’s disease. Audiol. Neurootol..

[23] Salviz M., Yuce T., Karatas A., Balikci H.H., Ozkul M.H. (2015). Diagnostic value of frequency-associated vestibular-evoked myogenic potential responses in Ménière’s disease. Audiol. Neurootol..

[24] McCaslin D.L., Fowler A., Jacobson G.P. (2014). Amplitude normalization reduces cervical vestibular evoked myogenic potential (cVEMP) amplitude asymmetries in normal subjects: Proof of concept. J. Am. Acad. Audiol..

[25] Jha R.H., Piker E.G., Gomez J. (2024). Effects of Age on the Frequency Amplitude Ratio of Cervical and Ocular Vestibular Evoked Myogenic Potentials. Am. J. Audiol..

[26] Dabbous A.O., El Bohy Z., Helal S., Hamdy H.S. (2023). Age effects on frequency amplitude ratio of cVEMP. Egypt. J. Otolaryngol..

[27] Singh N.K., Kumar P., Jagadish N., Mendhakar A., Mahajan Y. (2023). Utility of Inter-Frequency Amplitude Ratio of Vestibular-Evoked Myogenic Potentials in Identifying Meniere’s Disease: A Systematic Review and Meta-Analysis. Ear Hear..

[28] Jerin C., Berman A., Krause E., Ertl-Wagner B., Gürkov R. (2014). Ocular vestibular evoked myogenic potential frequency tuning in certain Menière’s disease. Hear. Res..

[29] Sobhy O.A., Elmoazen D.M., Abd-Elbaky F.A. (2019). Towards a new staging of Ménière’s disease: A vestibular approach. Acta Otorhinolaryngol. Ital..

[30] Taylor R.L., Welgampola M.S., Nham B., Rosengren S.M. (2020). Vestibular-Evoked Myogenic Potential Testing in Vestibular Localization and Diagnosis. Semin. Neurol..

